# Vision screening in children: Is 7-9 years of age a threshold for visual impairment?

**DOI:** 10.12669/pjms.325.10367

**Published:** 2016

**Authors:** Yusuf Haydar Ertekin, Murat Tekin, Aysegul Uludag, Sedat Arikan, Erkan Melih Sahin

**Affiliations:** 1 Yusuf Haydar Ertekin, Department of Family Medicine, Canakkale Onsekiz Mart University School of Medicine, Canakkale 17042, Turkey; 2 Murat Tekin, Department of Family Medicine, Canakkale Onsekiz Mart University School of Medicine, Canakkale 17042, Turkey; 3 Aysegul Uludag, Department of Family Medicine, Canakkale Onsekiz Mart University School of Medicine, Canakkale 17042, Turkey; 4 Sedat Arikan, Department of Ophthalmology, Canakkale Onsekiz Mart University School of Medicine, Canakkale 17042, Turkey; 5 Erkan Melih Sahin, Department of Family Medicine, Canakkale Onsekiz Mart University School of Medicine, Canakkale 17042, Turkey

**Keywords:** Eyeglasses, Strabismus, Vision disorders, Vision screening, Visual acuity

## Abstract

**Objective:**

The present study aimed to assess the prevalence of decreased visual acuity, strabismus, and spectacle wear in children aged 5 to 13 years.

**Methods:**

A cross-sectional study was performed in primary education schools. A total of 1938 participants, including 940 females (48.5%) and 998 males (51.5%) with a mean age 8.96 ± 2.31 (5-13 years old), were screened. The comparisons were performed with gender, age, and age groups. The children attended to vision screening were assigned to three age groups as 5-6 years, 7-9 years, and 10-13 years.

**Results:**

The prevalence of the parameters was detected as decreased visual acuity 12.4%, strabismus 2.2%, and spectacle wear 6.9%. The prevalence of decreased visual acuity was significantly higher in girls and in children aged 7-9 years old (p = 0.013, p < 0.001). The prevalence of spectacle wear was significantly higher in girls and in children aged 7-9 years old (p = 0.019, p < 0.001). There was a visual acuity decrease in 33 of 106 (31.1%) children despite wearing own spectacle. There was no significant difference among three age groups for strabismus.

**Conclusion:**

Increased prevalence of decreased visual acuity, as well as the higher frequency of spectacle wear in children at ages of 7-9 years old may point out a threshold for visual impairment.

## INTRODUCTION

Early diagnosis and treatment, through vision screening, provide significant clinical benefits.^1^ According to the last guideline of the American Academy of Pediatrics (AAP), vision assessment is recommended in each well-child visit from newborn term until the child is three years old.^2^ Therefore, three years old children or older ones have been suggested to get a proper vision screening or a measurement of visual acuity.^1^ Moreover, AAP / Bright Futures guidelines suggest vision screening after school entry at 5, 6, 8, 10, 12, 15, and 18 years.^3^

Effectiveness of vision screening practices in children has been associated with the ratio of detecting the preventable potential vision problems, such as amblyopia, leading to younger age related visual impairment.^4^ The critical age limit for getting favorable results in the management of amblyopia such as improved visual acuity has been suggested up to 7-9 years of age.^5^ Despite the routine eye screening, it was reported that 1/20 of children may be at risk of permanent visual impairment resulting from amblyopia or strabismus in the United States.^6^ At the same time, strabismus can give rise to complications such as amblyopia, diplopia, and psychosocial issues in school-age children.^6^

Despite having potential effective results of vision screening, the United States Preventive Services Task Force has reported the insufficient evidence, indicating new researches for vision screening application for asymptomatic school children and adolescents. Because of the limited data related to the investigation of the prevalence of children older than five years old with visual impairment,^7^ we aimed to perform vision screening program consisting of visual acuity measurement and strabismus examination for children above five years of age.

## METHODS

This cross-sectional-descriptive study was conducted between September and December of 2013. The population of this study consisted of 7579 children above five years of age, who were in primary education school in the city center of Canakkale, Turkey. According to the cluster sampling, the city center was divided into the five clusters. One primary education school was randomly selected from each cluster in the city center of Canakkale. Therefore, five schools were selected from five clusters. All classes in the mentioned schools, as well as all students from these classes were enrolled in this study. The sample size was calculated using a sampling formula under known conditions as a minimum of 366 individuals. The study organization was prepared base on 2960 children in these five schools. Approximately 65.5% (n=1938) of students of five schools were able to participate in the present study because of various reasons such as absence of the students in the school on the date of vision screening program or unwillingness of the parents to participate their children in this program.

## Data analysis and definitions

Data was collected using a family information form and a student examination form, which were developed by the investigators. A preliminary application was performed in a sub-sample of similar participants and the deficiencies of the screening organization that are distance and sitting level to chart/book, light sources and intensity, validation of the physician’s diagnosis was eliminated.

Visual acuity was measured in a prepared room with adequate light at 6 m distance using a 10-row mixed decimal eye chart. For 10-row eye chart, the range of scores was 10/10 for the largest row to 1/10 for the smallest one. Visual acuity score <8/10 (0.1 logMAR) (<6/10 for children under 6 years of age) or ≥3 lines difference between two eyes, because this may be an indicator of amblyopia was accepted as decreased visual acuity cut-off for referral.^8^-^10^ Strabismus was examined using cover/uncover test and the Hirschberg test.^11^

Physician-prescribed spectacle wear was questioned by family information form and supported by children’s statement. Children with spectacle were evaluated with their own spectacles. Children who were not keeping own spectacle at that moment were evaluated with the specific condition noted.

All physicians, which are family physician-physician assistants were trained by a single ophthalmologist. Each test was performed by the same physician from the beginning to the end of the vision screening program under the supervision of an ophthalmologist.

## Statistical analysis

A commercial software (SPSS, ver. 19.0; SPSS, Inc., Chicago, IL) was used in the data analysis. Prevalence rates were given as the ratio of the number of participants with related visual disorder to the total participants calculated. The means, standard deviations, and percentages were calculated for descriptive purposes. Pearson’s chi-square with 95% confidence interval and pairwise post hoc analyses was used to assess the statistical significance. A p value of less than 0.05 (two-sided) was accepted as statistically significant.

## Ethics statement

This study was certified by the University of Canakkale Onsekiz Mart Research Ethics Committee and the Provincial Directorate of National Education. The study was conducted in accordance with the principles of the Declaration of Helsinki. Written consent was obtained from the parents of all children, after a clarification of the nature and possible results of the study prior to the examination.

## RESULTS

A total of 1938 participants, including 940 females (48.5%) and 998 males (51.5%) with a mean age 8.96 ± 2.31 (5-13 years old), were screened. The prevalence rates, including newly recognized cases are shown by age and gender in Table-I.

**Table-I:** Prevalence rates and gender distribution by age and gender, n (%).

		*Gender*		*Decreased visual acuity*		*Strabismus*		*Spectacle wear*

*Age*	*Total*	*Female*	*Male*	*Prevalence*	*Newly recognized*	*Prevalence*	*Newly recognized*	*Prevalence*
5	75 (3.9)	39 (52.0)	36 (48.0)	3 (4.0)	1 (1.3)	1 (1.3)	1 (1.3)	2 (1.5)
6	401 (20.7)	202 (50.4)	199 (49.6)	37 (9.2)	23 (5.7)	12 (3.0)	6 (1.5)	14 (10.5)
7	350 (18.1)	163 (46.6)	187 (53.4)	47 (13.4)	24 (6.9)	6 (1.7)	4 (1.1)	23 (17.3)
8	277 (14.3)	132 (47.7)	145 (52.3)	43 (15.5)	14 (5.1)	9 (3.3)	6 (2.2)	29 (21.8)
9	255 (13.2)	143 (56.1)	112 (43.9)	45 (17.6)	9 (3.5)	6 (2.4)	3 (1.2)	36 (27.1)
10	147 (7.6)	62 (42.2)	85 (57.8)	12 (8.2)	5 (3.4)	1 (0.7)	1 (0.7)	7 (5.3)
11	149 (7.7)	70 (47.0)	79 (53.0)	18 (12.1)	11 (7.4)	2 (1.3)	0 (0.0)	7 (5.3)
12	138 (7.1)	70 (50.7)	68 (49.3)	18 (13.0)	10 (7.2)	2 (1.4)	1 (0.7)	8 (6.0)
13	146 (7.5)	59 (40.4)	87 (59.6)	18 (12.3)	11 (7.5)	4 (2.7)	3 (2.1)	7 (5.3)

Total	1938	940 (48.5)	998 (51.5)	241 (12.4)	108 (5.6)	43 (2.2)	25 (1.3)	133 (6.9)

The prevalence of decreased visual acuity, strabismus, and spectacle wear were 12.4% (CI: 10.9 – 13.9), 2.2% (CI: 1.6 – 2.9), and 6.9% (CI: 5.7 – 8.0), respectively. At the same time, 108 (5.6%, CI: 4.6 – 6.6) children with decreased visual acuity and 25 (1.3%, CI: 0.8 – 1.7) children with strabismus was newly recognized.

Although no difference among gender was found according to strabismus, the prevalence of decreased visual acuity and spectacle wear was significantly higher in girls compared to boys (Table-II).

**Table-II:** Vision disorders and spectacle wear by gender, n (%) [Confidence Interval].

	*Female*		*Male*		*p*
Decreased visual acuity	135 (14.4)	[12.1 – 16.6]	106 (10.6)	[8.7 – 12.5]	0.013
Strabismus	20 (2.1)	[1.2 – 3.1]	23 (2.3)	[1.4 – 3.2]	0.878
Spectacle wear	78 (8.3)	[6.5 – 10.0]	55 (5.5)	[4.1 – 7.0]	0.019

The highest ratio of decreased visual acuity was detected in children aged 7, 8, and 9 years old. Therefore, the participants were assigned to three age groups for comparison as 5-6 (Group 1), 7-9 (Group 2), and 10-13 years old (Group 3).

## Comparisons of prevalence rates according to the age groups

The prevalence of decreased visual acuity in Group 1, Group 2, and Group 3 was found to be 8.4% (n=40), 15.3% (n=135), and 11.4% (n=66), respectively (p < 0.001). The post hoc analysis revealed that Group 2 had a significantly higher prevalence of decreased visual acuity in comparison to Group 1 (p < 0.001) and Group 3 (p = 0.036). However, there was no significant difference between Group 1 and Group 3 (p = 0.123).

The prevalence of strabismus in Group 1, Group 2, and Group 3 was calculated as 2.7% (n=13), 2.4% (n=21), and 1.6% (n=9), respectively. There was no significant difference among groups (p = 0.392).

The prevalence of spectacle wear was significantly higher in Group 2 (n=88, 10.0%, CI: 8.0 – 12.0) than Group 1 (n=16, 3.4%, CI: 1.7 – 4.9) and Group 3 (n=29, 5.0%, CI: 3.2 – 6.8) (p < 0.001).

## Decreased visual acuity in children with spectacle

Among 133 children declared to wear spectacle, of 106 children were evaluated with their own spectacles, and of 27 were evaluated without spectacle because they have not spectacle along at the time of the vision screening. There was a visual acuity decrease in 33 of 106 (31.1%) children despite wearing own spectacle. Twelve of 27 (44%) children who were assessed without spectacle during the examination had a decreased visual acuity.

## DISCUSSION

The present study demonstrated the prevalence of decreased visual acuity that was detected in children with 5-13 years of age to be 12.4%. This data of the present study is consistent with the result of the similar studies in which decreased visual acuity prevalence in the same age range of the children was found as 9-25.7%.^12^-^15^ Some of the differences in prevalence between countries are known to be caused by ethnicity.^16^ In particular, higher rates of refractive error in Chinese and Hispanic populations have been demonstrated in a study comparing six ethnic structure.^17^ However, apart from these reports, very low value of prevalence rate can also be found in the literature, such as Unsal et al. have notified the prevalence of decreased visual acuity in children aged 6-17 years to be 1.7% in Turkey.^18^ In their study, different from the present study, they had accepted cut-off value of decreased visual acuity score for referral in children aged 6-17 years as <5/10. But this cut-off value is known to be appropriate for the diagnosis of low visual acuity in children 48–60 month-old age group.^19^ Pan et al. have revealed the proportionally increase in cut-off values for decreased visual acuity in children aged 30 to 60 months corresponding to 20/63 to 20/40 Snellen equivalent (3/10 to 5/10), and they attributed the determination of the cut-off value for decreased visual acuity according to age-groups to cooperation level, improvement of cognitive abilities, or to maturation of visual function itself.^9^ On the basis of this suggestion, while evaluating the low visual acuity in childhood, it would be better if cut-off value of decreased visual acuity was adjusted with taking age groups into consideration. Therefore, in the present study, a cut-off value of 5/10 for 5-aged children was used, that is different from the older children whose cut-off value was established as 8/10.

Visual acuity in children aged 5-13 was investigated in accordance with the age rather than age groups in related studies.^13^,^20^ In two of these studies, it was shown that the prevalence of myopia, which is closely associated with low visual acuity, was demonstrated to increase proportionally with age. In a previous study conducted by Zhao et al., initiation of myopic refractive error has been suggested to occur in children aged 8 years old.^20^ Moreover, Chung et al. reported the rate of referred myopic children aged 9-10 years old as 42%.^13^ Similar with these studies, in the present study, the prevalence of decreased visual acuity was detected at the highest rate in children aged 7, 8, and 9 years old which were accepted as limit ages for treatment of amblyopia. So that, determining the children with refractive error in ages between 7-9 years old appears to be beneficial for preventing amblyopia.

The current study also revealed a higher rate of spectacle wear in Group 2 (7-9 years old) when compared with Group 1 (5-6 years old) and Group 3 (10-13 years old). To the best of our knowledge, there are no studies evaluating the ratio of spectacle wear in children according to age groups, thus this study could be one of the first in this context. The studies investigating the rate of spectacle wear in children are limited in the literature.^16^ However, Aldebasi et al. performed a study in the similar age group (6-13 years old), they determined the rate of spectacle wear as 5.5% during vision screening, however different from the present study, and they did not query the presence of physician-prescribed spectacle in overall children with low visual acuity in their study.^21^ The cause of lower detection rate of spectacle wear in the present study may be related to many situations linked with barriers like appearance, being teased by peers, costs etc for non-compliance reported by Sharma et al.^16^

The prevalence of strabismus in children has been reported to vary between 2% and 4%.^15^,^22^-^25^ Inconsistent with these results, the prevalence of strabismus was found to be 2.2% in the current study. As it was previously suggested in the study of Lanca et al., the prevalence of strabismus did not differ according to age groups in the present study.^15^ In another study, Kvanström et al. retrospectively examined the children from birth to ten years of age, and they demonstrated the peak age of strabismus as 4 years old, along with the detection of very few cases in children above 4 years old.^22^

The strengths of the study are the considerable sample size and efforts of the children and their parents, the professional support of the ophthalmologist to gather accurate ophthalmic data and the usage of the proper cut-off values.

### Limitations of the study

This study is limited by the lack of best corrected visual acuity and thus do not report amblyopia rates.

## CONCLUSION

Decreased visual acuity and strabismus affects one in eight children aged 5 to 13 years old. Our results highlights that 7-9 years of age is a significant period in children for observing visual impairment. Almost half of children with physician-prescribed spectacle may not have sufficient visual acuity. More researches are needed to assess children according to age groups for vision problems to investigate new strategies.
